# Functional Head Impulse Testing Might Be Useful for Assessing Vestibular Compensation After Unilateral Vestibular Loss

**DOI:** 10.3389/fneur.2018.00979

**Published:** 2018-11-19

**Authors:** Julia Sjögren, Per-Anders Fransson, Mikael Karlberg, Måns Magnusson, Fredrik Tjernström

**Affiliations:** Department of Clinical Sciences Lund, Faculty of Medicine, Skåne University Hospital, Lund University, Lund, Sweden

**Keywords:** vHIT, DVA, vestibulo-ocular reflex, vestibular loss, vestibular rehabilitation

## Abstract

**Background:** Loss of the vestibulo-ocular reflex (VOR) affects visual acuity during head movements. Previous studies have shown that compensatory eye-saccades improve visual acuity and that the timing of the saccade is important. Most of the tests involved in testing VOR are made with passive head movement, that do not necessarily reflect the activities of daily living and thus not being proportionate to symptoms and distresses of the patients.

**Objective:** To examine differences between active (self-generated) or passive (imposed by the examiner) head rotations while trying to maintain visual focus on a target.

**Method:** Nine subjects with unilateral total vestibular loss were recruited (4 men and 5 women, mean age 47) and tested with video Head Impulse Test (vHIT) and Head Impulse Testing Device-Functional Test (HITD-FT) during passive and active movements while looking at a target. VOR gain, latencies of covert saccades, frequency of covert saccades and visual acuity were measured and analyzed.

**Results:** Active head-impulses toward the lesioned side resulted in better visual acuity (*p* = 0.002) compared to conventional passive head-impulses and generated eye-saccades with significantly shorter latencies (*p* = 0.004). Active movements to the lesioned side generated dynamic visual acuities that were as good as when testing the intact side.

**Conclusion:** Actively generated head impulses resulted in normal dynamic visual acuity, even when performed toward the side of total vestibular loss. This might be attributed to the appearance of short-latency covert saccades. The results show a strong relationship between self-generated movements, latencies of covert saccades and outcome in HITD-FT, i.e., a better dynamic visual function with less retinal slip which is the main function of the VOR. The method of active HITD-FT might be valuable in assessing vestibular compensation and monitoring ongoing vestibular rehabilitation.

## Introduction

The vestibulo-ocular reflex (VOR) has an important function in stabilizing gaze, i.e., keeping the visual target on the fovea during movements of the head to maintain visual acuity (1). After a chronic unilateral vestibular loss (uVL), the reflex is impaired and can clinically be tested with the head-impulse test (HIT). This bedside test is quick and easy to perform and consists of a quick low amplitude rotation of the patient's head, stimulating the semi-circular canals in the plane of the movement. When performed toward the side of the vestibular lesion, the eyes will lag due to reduced vestibular input. This will cause the gaze to follow the direction of the head, instead of being locked on the target. When perceived, a corrective saccade is generated, disclosing the impairment (2). Due to central vestibular compensation however, the corrective eye-saccade does not always follow after the head movement but can start while the head is still moving. Such a saccade is impossible to detect by the observer (3) but can be recorded with search coils or video-oculography (1, 4). A saccade that occurs while the head is still moving is defined as a covert saccade and a saccade that occurs after the head has stopped moving as an overt saccade (1, 5). Although the mechanism of covert saccades is unknown, there are studies that suggest that the low latency of covert saccades is triggered either by retinal slip (i.e., when the image on the retina moves away from the fovea and is thus not in focus (6), by somatosensory cues from the cervical segment (7), by possible residual labyrinthine function, by other cues that the head is about to or has just begun to rotate (8) or by generated internal models (i.e., preformed saccades that are triggered by certain movements with little or no feedback information) (9).

Peripheral vestibular semi-circular canal function is conventionally tested, by measuring the gain of the angular VOR (aVOR). A reduced gain together with a corrective eye-saccade is universally accepted as a sign of VOR hypofunction (10). Vestibular function can also be evaluated with so called “functional testing,” i.e., how well the aVOR performs with respect to its goal of stabilizing gaze in space (11). Those tests measure how well a subject acquire visual information during a head movement and reflects the combined effects of VOR and catch-up saccades on dynamic reading ability (12, 13). Due to their short-latencies, covert saccades may in particular reduce blurred vision (oscillopsia) and improve visual performance (14). The test however is generally performed with passive head movements, i.e., an examiner rotates the subject's head. Most movements in everyday life consist of active voluntary self-movements which are also encouraged by the general concept of vestibular rehabilitation, that invariably consists of training the brain to an absent VOR. Thus, the main aim of this study is to assess how well patients manage to maintain visual fixation during active head movements.

## Methods

### Subjects

Nine subjects (4 men and 5 women) with a mean age of 47 years (range 40–50 years), were recruited for the study: eight with unilateral vestibular loss after translabyrinthine surgery for vestibular schwannoma, with a mean time since surgery of 8 years (range 1–16 years) and one with congenital unilateral vestibular loss, probably due to an intrauterine cytomegalovirus infection. The total unilateral vestibular loss was confirmed by bi-thermal caloric tests, video head-impulse test of all six semi-circular canals and cervical vestibular evoked myogenic potentials.

### Ethical approval

The experiments were performed in accordance with the Helsinki declaration and approved by the local ethical board (Dnr 2016/32, EPN, Lund University, Sweden). All subjects gave their written and informed consent prior to participation.

### Experimental protocol

The ability to maintain focus on a visual reference point while performing fast rotational accelerations and decelerations of the head in the horizontal plane were assessed in all subjects using two methods. (1) The ability to control the eye movements so the focus did not deviate from a fixed visual reference point during fast accelerations and decelerations of the head, was assessed using an Interacoustics video-head impulse test (vHIT) system (EyeSeeCam version 1.2) (9). (2) The reading performance during fast rotational accelerations of the head was assessed with the Head Impulse Testing Device-Functional Test (HITD-FT) (15). All procedures were performed by the same examiner.

#### vHIT testing

The subject sat in an arm-less chair 1.5 m in front of a white wall facing an attached 3 × 3 cm blue marker placed at eye level, which served as visual focusing point during the head movement tests. The subject was instructed to keep focusing on the visual target on the wall at all times during the investigations. In all tests the same examiner stood behind the subject, then started to impose manual fast rotational horizontal head movements with peak velocities exceeding 150°/s, accelerations/decelerations chiefly within 3,000–8,000°/s^2^ and a movement amplitude of about 10–20°. The head movement testing continued until the Interacoustics software had accepted the performance of at least 10 passive head movement recordings in each direction. After the conventional passive head movement testing, the subject was asked to perform active horizontal head movements of similar velocity, acceleration, and amplitude as used when assessing the performance during passive head movements. The subjects were allowed to train a few times before the actual test. The head movement testing continued during active head movements until the Interacoustics software had accepted the performance of at least 10 head movement recordings in each direction (i.e., the head movement performance criteria imposed by the software were identical during active and passive head movements). The Interacoustics system record eye and head movements at a sample frequency of 220 Hz, whilst the analyses of the recorded data were based on signal-processed data elevated to the sampling frequency of 1,000 Hz by the software.

#### HITD-FT testing

The subjects were placed 1.5 m from the HITD-FT computer monitor. The patient was wearing a head mounted accelerometer. Static visual acuity was first evaluated using an eye chart displayed on the monitor with letter sizes scaled according to the subject's viewing distance. The size of the visual stimuli used during the test was then determined based on the assessed visual acuity by increasing that of the smallest line seen by 0.8 log-MAR. This stimulus size, which is eight lines bigger than the best static visual acuity, would then remain constant during the test. The examiner manually imposed horizontal head-impulses, a minimum of 10 valid impulses in each direction. The HITD-FT software had the same criteria for a valid head-impulse during active and passive head movements, the impulses had to reach an acceleration between 3,000 and 6,000°/s^2^. When the imposed head angular acceleration exceeded a user-defined threshold, a Landolt's C optotype in one of eight possible orientations was briefly displayed on the screen. The HITD-FT monitor was full HD (1,920 × 1,080 resolution) with a maximum response time of 5 ms and a refresh rate of 60 Hz. The duration of the letter display was set to two frames, this resulted in the optotype being visual during about 33 ms with no delay (16) which results in the visual display being visible around the time of maximum head acceleration.

#### Data analysis

The vHIT performance during passive and active head movements were assessed by both the Interacoustics software and a customized software. The Interacoustics software determined the average gain (i.e., the quotient between average eye velocity and head velocity) during the head acceleration phase (0–100 ms). The customized software determined whether the eye movement recordings included saccades, and if so, the time from when head movement started until the first saccade reached peak velocity (denotes peak latency). The software also determined if the saccade started while the head still moved in the initial movement direction (Covert) or after the head movement had stopped or had started to move in the reverse direction (Overt). The start of the head movement was defined to be when the head movement velocity exceeded 30°/s. An eye movement was defined as a saccade if the eye peak velocity exceeded 80°/s, if both the acceleration and deceleration flanks exceed 3,000°/s^2^ and if the saccade duration was within 10–80 ms. The HITD-FT data was calculated by the fHIT 1.0 system (16). The proportion of head impulses that generated covert saccades was measures in percentage.

#### Statistical analysis

The parameters HITD-FT score, vHIT gain, saccade peak latency, and the proportion of covert/overt saccades made, were analyzed using repeated measures GLM ANOVA (General Linear Model Analysis of Variance) (17). The main factors and factor interactions analyzed were: “Active/Passive” (Active vs. Passive head movements; d.f. 1); “Ipsi/contralesional” (Ipsilesional vs. Contralesional head movement direction; d.f. 1). When analyses of some parameters revealed significant main factor interactions, repeated measures GLM ANOVA analyses were performed on all parameters with only one main factor “Active/Passive” (Active vs. Passive head movements; d.f. 1) individually on ipsilesional and contralesional data.

The Wilcoxon matched-pairs signed-rank test (Exact sig. 2-tailed) was used for within-group *post-hoc* comparisons, i.e., analyzing the difference between ipsilesional and contralesional head movement responses during active and passive head movements (17).

In all analyses, *p*-values < 0.05 were considered significant. Non-parametric statistical tests were used in all statistical evaluations since the Shapiro-Wilk test revealed that some data sets were not normally distributed and normal distribution could not be obtained by log-transformation. The repeated measures GLM ANOVA analysis was used after ensuring that all model residuals had normal or approximate normal distribution (17).

Sample size analyses, using the statistical package G-power™, were performed on the parameters used that were unaffected by boundaries (i.e., the HITD-FT and the % Covert saccades parameters could only assume values within the range of 0–100). The sample size analysis of the vHIT gain parameter revealed an effect size of 1.8, which shows that with the *p*-value set to 0.05 (2-tailed), our study would require *n* = 5 subjects to reach a power value of 0.8 for this parameter. The sample size analysis of the saccade peak latency parameter revealed an effect size of 2.2, which shows that with the *p*-value set to 0.05 (2-tailed), our study would require *n* = 4 subjects to reach a power value of 0.8 for this parameter.

The statistical analyses were performed with SPSS version 24 and the power analysis was performed with GPower 3.1.

## Results

**Figure 1:**
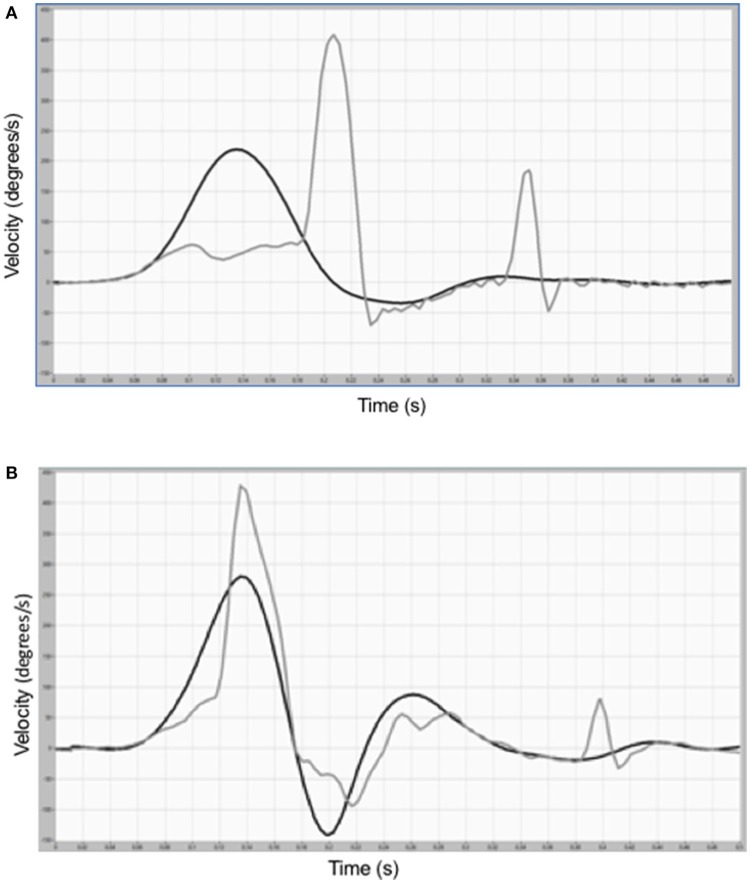
**(A)** Eye and head velocity traces during a conventional, passive head-impulse test toward the ipsilesional side in one representative subject show low eye velocity during the head movement and late covert saccade (after 135 ms) and an overt compensatory saccade. **(B)** Eye and head velocity traces in the same subject during an active head rotation toward the ipsilesional side show a large early (after 60 ms) covert saccade that occurs during the head movement.

Passive and active eye and head velocity traces from a subject during conventional HIT toward the ipsilesional side are shown in Figure 1. During passive HIT there are one late covert and one overt saccade after the head movement (Figure 1A), but during active HIT there is a large early covert saccade during the head movement (Figure 1B).

Table 1 shows the mean HITD-FT-score, mean gain, mean peak saccade latency, and the mean frequency of impulses that generated a covert saccade for impulses toward the lesioned side and toward the contralesional side.

**Table 1:** Recorded performance during head-impulses in ipsilesional and contralesional directions.

		**HITD-FT**		**VOR gain**		**Saccade latency**		**% Covert saccades**	
		**Mean (%)**	**SEM**	**Mean**	**SEM**	**Mean(s)**	**SEM**	**Mean (%)**	**SEM**
Ipsilesional	Passive	64.4	8	0.34	0.02	0.134	0.015	77	12
	Active	91.2	3.1	0.54	0.06	0.085	0.009	99	1
Contralesional	Passive	93.7	3.5	0.92	0.03	0.233	0.031	27	13
	Active	91.1	4.2	0.89	0.05	0.208	0.025	35	12

**Figure 2:**
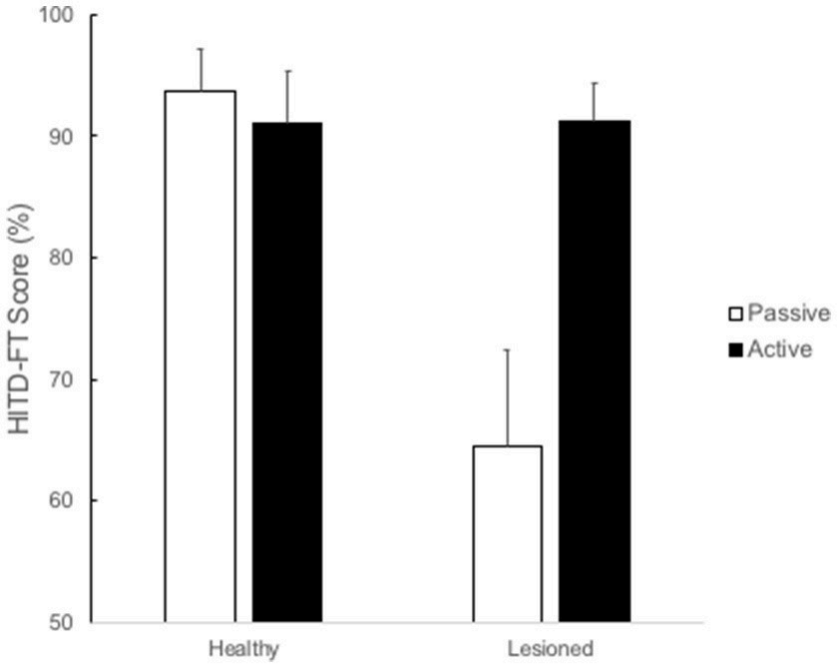
HITD-FT (%) rate of correct answers (%) in correlation to passive (white) or active (black) head-impulses toward the ipsi and contralesional side. Active head-impulses resulted in a better HITD-FT score (91.2%) compared to passive head-impulses (64.4%) *p* = 0.002.

The outcome in HITD-FT was influenced by whether the test was active or passive (*p* = 0.021) and toward which side the test was performed (*p* = 0.008; Table 2). The greatest increase of HITD-FT score during active movements was toward the lesioned side (*p* = 0.002, Table 3) as is demonstrated in Figure 2. Figure 2 also shows that during active head movements, no difference can be seen in performance regardless of whether the head movement was executed toward the ipsilesional or contralesional side.

**Table 2:** Repeated measures GLM-ANOVA analyses of HITD-FT scores, gain, saccade latency and frequency of covert saccades, determining if the performance depended on active/passive head impulses and on ipsilesional/contralesional head movement directions.

		**Active/Passive**	**Ipsi/Contralesional**	**Active/Passive + Ipsi/Contralesional**
HITD-FT	*p*-value	**0.021**	**0.008**	**0.005**
	*F*-value	8.1	12.1	15.2
VOR gain	*p*-value	**0.006**	<**0.001**	**0.015**
	*F*-value	13.7	90.2	9.5
Saccade latency	*p*-value	**0.048**	**0.001**	0.372
	*F*-value	5.4	23.5	0.9
% Covert saccades	*p*-value	0.104	**0.001**	0.725
	*F*-value	3.4	23.5	0.1

*Bold statistics denote p-values < 0.05*.

**Table 3:** Repeated measures GLM-ANOVA analyses of the performance during active vs. passive head-impulses when the head movements were performed in ipsilesional and contralesional directions.

**Active vs. Passive**	**Contralesional**		**Ipsilesional**	
	***p*-value**	***F*-value**	***p*-value**	***F*-value**
HITD-FT	0.638	0.2	**0.002**	19.6
VOR gain	0.370	0.9	**0.005**	14.4
Saccade latency	0.387	0.8	**0.004**	16.4
% Covert saccades	0.303	1.2	0.084	3.9

*Bold statistics denote p-values < 0.05*.

The gain values increased significantly when the head movements were active (*p* = 0.006; Table 2) and separated GLM ANOVA analyses show that for head movements toward the ipsilesional side there was significant change (*p* = 0.005, Table 3). Saccade latency was significantly shorter (*p* = 0.048, Table 2) when performing active head movements, especially toward the ipsilesional side (*p* = 0.004, Table 3).

The percentage of generated coverts during head movements are shown in Table 1. Although the frequency of coverts increased during active movements, it was not statistically significant (Tables 2, 3). Table 4 shows post-hoc Wilcoxon analyses of HITD-FT scores, gain, saccade latency, and frequency of covert saccades.

**Table 4:** *Post-hoc* Wilcoxon analyses of HITD-FT scores, gain, saccade latency, and frequency of covert saccades.

		**Ipsilesional/Contralesional**
HITD-FT	Passive	**0.008**
	Active	1.0
VOR gain	Passive	**0.004**
	Active	**0.012**
Saccade latency	Passive	**0.004**
	Active	**0.004**
% Covert Saccades	Passive	**0.008**
	Active	**0.016**

*Bold statistics denote p-values < 0.05*.

## Discussion

**Figure 3:**
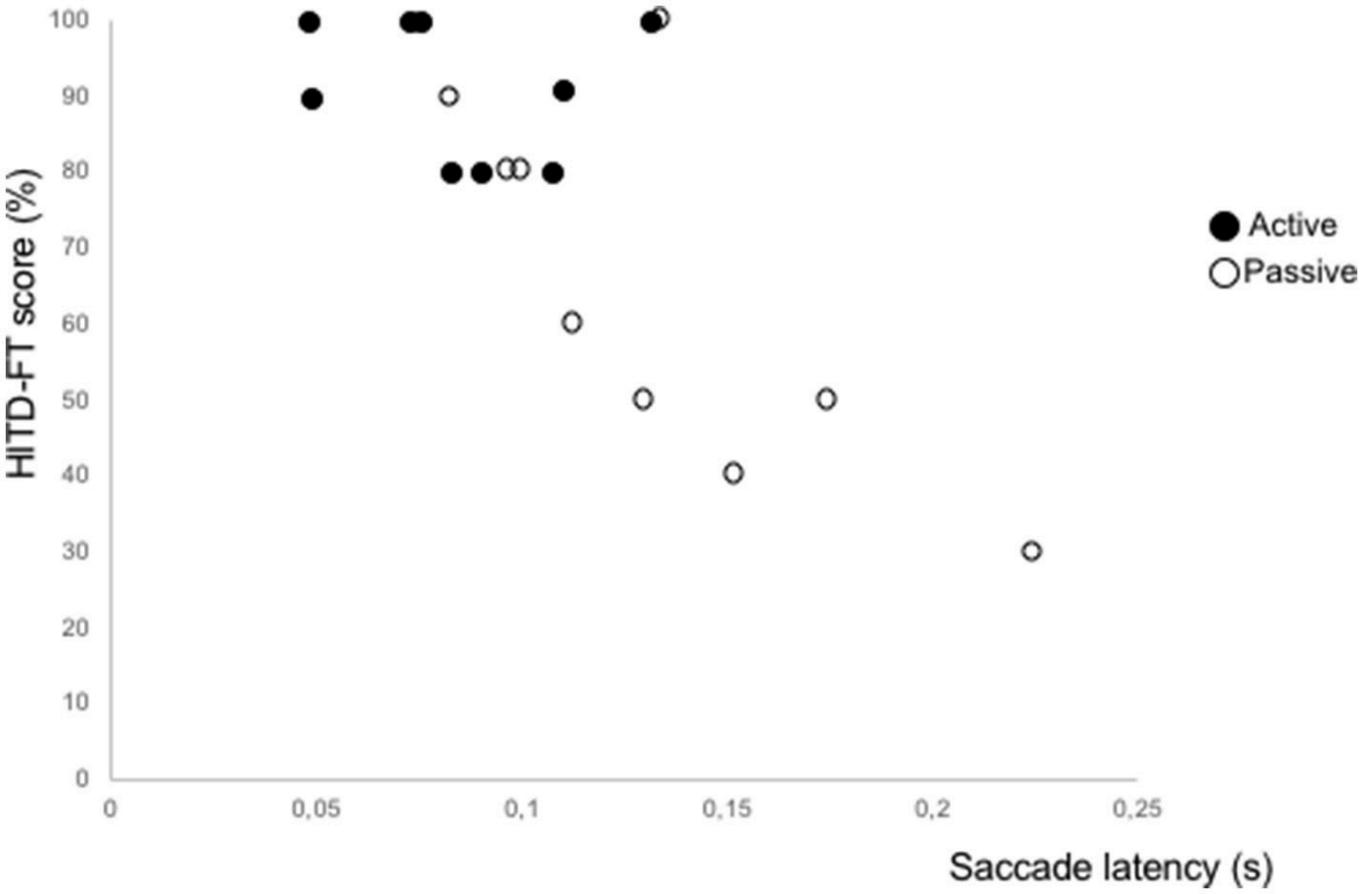
Graphical illustration of HITD-FT scores vs. latencies of corrective saccades, triggered during passive and active ipsilesional head-impulses.

The dynamic visual acuity was found to be better during active head impulses as compared to passive ones, and the main reason seems to be a shorter latency of the first covert saccade. Actively generated head movements in our subjects with long-standing uVLs, yield similar HITD-FT scores toward the contralesional and the ipsilesional side. This is consistent with previous findings of less difference in dynamic visual acuity during self-generated movements than passively imposed movements in patients with uVL (18). It has been shown that covert saccades are triggered earlier during active head turns (19, 20), and that the latency correlates to dynamic visual acuity (21), but the combination of active head movements and covert saccade with regard to dynamic visual acuity has not been examined before. Figure 3 shows the saccade latencies plotted against HITD-FT values during active and passive head movements. Since most of the scores during active head movements are close to 100%, statistical correlation analyses become problematic.

Vestibular rehabilitation has been shown to increase dynamic visual acuity during active movement (22), an effect attributed to increased VOR gain. This is consistent with the results of the present study but, depending on how the VOR gain is calculated, the gain increase might be an artifact generated by the covert saccades. Simplified, in the EyeSeeCam-device the gain is derived from head and eye velocity during the acceleration phase calculated with a regression model including saccades (23). If the covert saccade in the case of an active head movement is generated during the acceleration phase, it would mean by itself a higher gain value (Figures 1A,B). In the present study, however, we have not focused on gain calculations and other studies have found higher gain values during active than during passive movements (9, 19).

It has also been shown that a higher percentage of generated covert saccades are correlated with better visual acuity (13), though that has not been examined with regards to saccade latency. In the present study the percentage of covert saccades did increase when performing active movements, but not significantly so.

The main shortcoming of the present study is the relatively small sample size, with a relatively small age-span. This makes it difficult to do analyzes of subjective parameters as well as to generalize the results to other populations, e.g., the age-dependent presby-vestibulopathy that are also affected by other factors relevant to vestibular compensation. However, the results show a strong relationship between self-generated movements, latencies of covert saccades and outcome in HITD-FT, i.e., a better dynamic visual function with less retinal slip, which is the main function of the VOR. Most of vestibular rehabilitation exercises are designed to challenge a deficient VOR, in order to induce compensation (24). All patients in the present study (except the one with congenital uVL) performed our VOR exercises albeit some years previously. The group comprised only well-compensated patients. We considered the homogeneity of the group of importance in order to elucidate whether active and passive head impulses differ, both in ocular responses as well as in dynamic visual acuity. Future studies will have to compare the full range from acute onset of vestibular loss as well as fully compensated patients to establish whether active head impulses generate a satisfactory measure of compensation as well as could be used to monitor vestibular rehabilitation.

The generation of covert saccades seems to be part of central vestibular rehabilitation, and they are possibly related to better compensation after suffering vestibular loss, though subjective parameters seem to be difficult to correlate to HITD-FT results (12). Our material is too small to estimate subjective outcome. However, it could be argued that the active movements are physiologically more similar to everyday head movements (25), especially when moving actively as in walking. Our results suggest that HITD-FT with actively imposed impulses may be a better tool for assessing the effect of vestibular adaptation than conventional passive head-impulses. To our knowledge this has not been shown prior to this study. In addition to perhaps being a tool for assessing compensation it is also instructive for patients in the mechanism of gaze stabilization, further motivating them to continue with vestibular rehabilitation exercises. More prospective research is needed with larger cohorts in assessing the course of active HITD-FT over time after uVL preferably together with subjective estimates of vestibular function, such as self-reports, e.g., the Dizziness Handicap Inventory and Vertigo Symptom Scale.

The nature of the saccades generated in active vs. passive movements is intriguing and raise still more questions. Obviously in some cases both active and passive impulses triggered feed-back reflexes since they have relative constant latencies. However, some patients have saccades with active movements with close to no latency at all, which could be interpreted as having cortical origin (feed-forward mechanism). If the actively generated saccades are of cortical origin, why do some impulses yield saccades that have latencies at all? If they are triggered by the same reflex action, a possible relay station would be the vestibular nuclei. Another hypothetical explanation could be that when performing an active head movement to one side the inhibitory neurons of the saccadic system are in turn inhibited, thus making the system as such “trigger-happy” for releasing any saccade. Indeed, an elaborate mathematical model has been put forward (9) and further studies are needed to further study the nature and origin of saccades, as from a rehabilitation point of view they seem to be a desirable trait to acquire.

## Conclusion

Actively generated head impulses result in almost normal HITD-FT values even when performed toward the side of total vestibular loss in patients with chronic unilateral vestibular loss. This might be attributed to the appearance of short-latency covert saccades. The method of active HITD-FT might be valuable in assessing vestibular compensation and monitoring ongoing vestibular rehabilitation.

## Data availability statement

The raw data supporting the conclusions of this manuscript will be made available by the authors, without undue reservation, to any qualified researcher.

## Author contributions

FT and P-AF conceived the study, interpreted results and contributed to the manuscript. JS executed the study, interpreted results and contributed to the manuscript. MK and MM interpreted results and contributed to the manuscript.

### Conflict of interest statement

The authors declare that the research was conducted in the absence of any commercial or financial relationships that could be construed as a potential conflict of interest.
